# A Transendothelial Leukocyte Transmigration Model Based on Computational Fluid Dynamics and BP Neural Network

**DOI:** 10.3389/fbioe.2022.881797

**Published:** 2022-06-21

**Authors:** Qingjia Chi, Zichang Yang, Hua-Ping Liang

**Affiliations:** ^1^ Department of Engineering Structure and Mechanics, School of Science, Wuhan University of Technology, Wuhan, China; ^2^ State Key Laboratory of Trauma, Burns and Combined Injury, Department of Wound Infection and Drug, Daping Hospital, Army Medical University, Chongqing, China

**Keywords:** random forest, back propagation neural network, cox regression, prognostic prediction, immune infiltration

## Abstract

The mechanism of immune infiltration involving immune cells is closely related to various diseases. A key issue in immune infiltration is the transendothelial transmigration of leukocytes. Previous studies have primarily interpreted the leukocyte infiltration of from biomedical perspective. The physical mechanism of leukocyte infiltration remains to be explored. By integrating the immune cell transmigration computational fluid dynamics (CFD) data, the paper builds a time-dependent leukocyte transmigration prediction model based on the bio-inspired methods, namely back propagation neural networks (BPNN) model. The model can efficiently predict the immune cell transmigration in a special microvascular environment, and obtain good prediction accuracy. The model accurately predicted the cell movement and flow field changes during the transmigration. In the test data set, it has high prediction accuracy for cell deformation, motion velocity and flow lift forces during downstream motion, and maintains a good prediction accuracy for drag force. The two prediction models achieved the prediction of leukocyte transmigration in a specific microvascular environment and maintained a high prediction accuracy, indicating the feasibility and robustness of the BPNN model applied to the prediction of immune cell infiltration. Compared with traditional CFD simulations, BPNN models avoid complex and time-dependent physical modeling and computational processes.

## Introduction

The mechanism of immune infiltration involving immune cells is closely related to various diseases. A key issue in immune infiltration is the transendothelial transmigration of leukocytes (immune cells). Among them, numerous microvascular stenosis fragments smaller than the cell size and tiny pores on the vessel wall are the key factors hindering the infiltration of immune cells. Previous studies have primarily interpreted the infiltration of immune cells from a biomedical perspective, ignoring the physical role in immune regulation. This paper combines various machine learning algorithms and computational fluid dynamics (CFD) simulation methods to systematically analyze the immune cell infiltration mechanism associated with liver cancer from the perspectives of biomedicine and mechanics.

In recent years, physics-based modeling approaches have become widely used analytical tools in engineering and environmental systems (Willard et al., 2022). However, the deviation between the actual engineering process and the physical laws adopted in the physical model makes us need to approximate the real value, leading to errors (Yan et al., 2020). In addition, the physical model contains many parameters estimated from limited observational data, which further degrades the performance of the physical model (Günther et al., 2020). Machine learning (ML) methods based on neural network models automatically extract complex relationships from data, theoretically solving scientific problems in physical models (Bentley, 2014; Kelley et al., 2016; Biswas et al., 2018; Huang et al., 2021; Jiang et al., 2021). The introduction of ML and deep learning methods achieved a leap from “model-driven” to “data-driven” (Xue et al., 2015; Jiang et al., 2021; Liu et al., 2022). This saves us from knowing the specific equations between the data, and the algorithm will automatically derive the data to match the target value. In many respects, the prediction accuracy of this method far exceeds that of traditional modeling methods due to the coverage of the vast training dataset (Valsamis et al., 2017; Sun et al., 2022; Wu et al., 2022; Yun and). But even state-of-the-art neural network models can be non-generalizable in specific studies due to the large-scale demand for data (Karpatne et al., 2017; Zhao et al., 2022). Therefore, researchers began to explore combining physical models and advanced neural network models to achieve their complementary strengths (Ansari et al., 2020).

In this regard, the currently most widely used neural network model (BPNN) plays a critical role (Dai and MacBeth, 1997). The combination method of physical and neural network models to practical problems is mainly divided into four aspects: parameterization (S. and S. et al., 2019), solution of partial differential equations (Yong et al., 2020), derivation of governing equations (Teng et al., 2020) and inverse modeling (Henry and F. et al., 2015). In fluid mechanics, researchers have gradually realized the efficiency and accuracy of predictive models (Wang et al., 2021). The forward calculation of physical problems is to predict the next state of the system by using physical parameters such as temperature, deformation, mass, and spatial position of the system. In contrast, inverse modeling uses the output of the system to derive the physical parameters of the system. In this regard, many high-fidelity datasets describing fluid mechanics phenomena facilitate the application of neural network methods to fluid mechanics (Fei et al., 2020; Yong et al., 2020). In recent years, deep learning methods for biophysical problems have emerged (Almagrabi et al., 2021). This modeling paradigm of exploring the laws of physics using efficient deep learning tools is known as data-driven modeling. These studies include predicting average Navier-Stokes uncertainty regions in high Reynolds (*Re*) environments (Templeton, 2015) and prediction studies of cylindrical velocity fields with different *Re* using fused convolutional neural networks (Jin et al., 2018).

Abundant experimental and numerical simulation data can help researchers understand the flow around a circular object. We used machine learning in previous studies to examine the role of immune infiltration in various diseases (Chi et al., 2020; Yang et al., 2021; Xu et al., 2022). This paper tries to adopt a research method combining ML method and physical model and take immune cell transmigration as the research object. We used CFD simulation data to build a neural network method for immune cell transmigration prediction. This method enables data value to be used repeatedly and achieves second-by-second forecasts that traditional methods cannot reach. Among them, the bio-inspired BPNN model is adopted and the prediction performance of the neural network and its superiority compared to the CFD method are discussed.

## Materials and Methods

### The CFD Model

In the blood flow of the human body, the size of immune cells is generally between 7 and 15 μm (Luo et al., 2011). To simplify the calculations, we use a cell model with a diameter of 10 μm for benchmark simulations. The cell model consists of viscous incompressible cell fluid and linear elastic cell membrane. The cell membrane thickness is 0.4 μm, the elastic modulus is 100 Pa, and the Poisson’s ratio is 0.25 (Aubry et al., 2015). Here, we choose the elastic modulus range of 50–500 Pa to reflect the physiologically possible mechanical response of the cell membrane. Cell fluid and plasma were designated as homogeneous fluids with the same parameters, with a kinematic viscosity of 0.0008 Pa s. The density of immune cells and plasma is 1,000 kg/m3. The phenomenon of cells passing through pores generally occurs in the capillary or venule vascular environment, where the blood flow rate is generally controlled at *Re* between 0.01 and 0.2 (Haber et al., 2013). Therefore, *Re* = 0.1 was adopted as the baseline plasma flow field. To investigate the retention effect of microvascular stenosis on cell transport, we selected six sets of parameters with channel widths of 10, 12, 14, 16, 18, and 20 μm to establish a microvessel model containing stenotic pores (Vollmar et al., 2019).

The immune cell transmigration model established in this paper includes a cell droplet and a microvascular model with narrow pores. The initial position of the immune cell is located on the upper side of the pore, and it will passively deform and pass through the pores smaller than its size under the drive of the fluid. Although a 3D model would be more accurate, we chose a 2D model to simplify the calculations. Studies have shown that the model is insensitive to depth in cells entering narrow pores (Leong et al., 2011). At the same time, an axisymmetric model containing half of the computational domain was adopted to analyze the transmigration of cells. The hemisphere model composed of the blue and cyan areas in the figure is the immune cell model, composed of a uniform cytosol wrapped by a linear elastic film. Both cellular fluid and extramembrane plasma are considered homogeneous incompressible Newtonian fluids. This paper regards the plasma flow in microvessels as Poiseuille flow in circular conduits. The velocity of the flow field near the wall is relatively small, and the velocity from the near wall to the microvessel axis rises parabolically and reaches a maximum value. The velocity of the flow field is symmetrical about the microvascular axis. Immune cells are located at the axis of the microvessels and are subjected to a symmetrically distributed fluid shear force. In this paper, COMSOL’s “laminar flow” is used to quantify the fluid flow of cell fluid and plasma. Immune cells are elastically deformed under the drive of blood flow, and “solid mechanics” is used to calculate the deformation of the cell membrane. We used “Fluid-Structure Interaction” to calculate the force-displacement transfer at the fluid-structure interface. The rust-colored area in the figure is the blood vessel wall. This paper ignores its deformation and regards it as a rigid wall.

The CFD model in this paper mainly uses COMSOL multiphysics simulation software for numerical calculation. The governing equation used in the model calculation is the Navier-Stokes (N-S) equation,

$$\rho_{f}\left[\frac{\partial U_{f}}{\partial t}+\left(U_{f}.\nabla \right)U_{f}\right]=\nabla \left[\mu \nabla U_{f}+\mu {\left(\nabla U_{f}\right)}^{T}\right]+F-\nabla P$$ (1)

The boundary condition is a no-slip boundary, namely the blood flow velocity near the wall is zero. The PARDISO solver is used in the COMSOL solver setting, which uses the stiffness decomposition matrix inversion to solve the N-S equation and uses parallel computing to improve the solution efficiency. The Euler method is used for meshing in COMSOL, and the motion of the fluid-solid boundary is described using an arbitrary Lagrangian-Euler (ALE) method. Distortion of the mesh near the fluid-solid boundary can be solved by remeshing. The COMSOL multiphysics simulation software has meshing capabilities that meet the computational accuracy requirements, and all meshing is completed within the software.

The ALE coordinate system, independent of the Euler and Lagrange coordinate systems, is generally not completely fixed in space, nor is it completely fixed on the material node. And the ALE grid could achieve the appropriate motion.

The Navier-Stokes equation described by ALE is

$$\rho \left(v_{t\left[x\right]}+\left(c\cdot \nabla_{x}\right)v\right)=\nabla_{x}\cdot \sigma +\rho b$$ (2)

The mesh velocity described by ALE can be easily obtained

$$\hat{v}=v-\frac{\partial x}{\partial Χ}w$$ (3)

where w is the velocity of the solid.

The model is calculated in a two-dimensional environment, and the leukocytes can move along the axial and radial directions of the tube with two degrees of freedom.

The numerical precision of the calculation adopts the default settings of the software. CFD tasks are completed on different computers due to too many cases to be solved.

### Theoretical Description

The so-called Poiseuille flow is the flow of viscous fluid in a circular pipe. When the Reynolds number is less than 2000, the liquid flow in a straight circular pipe with equal cross-section is laminar flow. Poiseuile law

$$\text{Q}=\text{ π}r^{4}\Delta p/\left(8\eta L\right)$$ (4)

It describes the steady flow of an incompressible viscous fluid in a horizontal circular pipe, and the Reynolds number is not large. When the flow form is laminar flow, the flow rate *Q* and the pressure difference *Δp* at both ends of the pipe, the pipe radius *r*_*0*_, the pipe length *l* and the fluid, the relationship of viscosity coefficient η. Poiseuille’s law is an essential law of fluid dynamics. Poiseuille flow is one of the few cases where there is an analytical solution to the N-S equation, the *x*-axis is chosen to be on the vessel axis, and y is the radial coordinate measured outward from the tube axis. Both the circumferential and radial velocity components are zero, and the velocity component parallel to the tube axis is denoted as *u*, which depends only on *y*. At the same time, the pressure is constant in each cross-section. In this way, in the N-S equation expressed in cylindrical coordinates, only one axial equation is left, which simplifies to:

$$\text{μ}\left(\frac{d^{2}u}{dy^{2}}+\frac{1}{y}\frac{du}{dy}\right)=\frac{dp}{dx}$$ (5)

According to the no-slip boundary condition, y=R, u=0 . Available velocity distribution:

$$\text{u}\left(y\right)=-\frac{1}{4\mu }\frac{dp}{dx}\left(R^{2}-y^{2}\right)$$ (6)

It can be seen that the velocity from the proximal wall to the microvascular axis increases parabolically.

At *y* = 0, i.e., on the tube axis (the center of the vessel)

$$u_{m}=\frac{R^{2}}{4\mu }\left(-\frac{dp}{dx}\right)$$ (7)

### Basic Theory of Neural Network

The basic unit of the neural network algorithm is the single-layer perceptron model. The structure of the single-layer perceptron model is simple, and its components only have an input layer and a single output layer. The figure below represents a perceptron model with two input neurons and one output neuron node. *x*_*1*_ and *x*_*2*_ represent the stimulus to the neuron, that is, the input information. *w*_*1*_ and *w*_*2*_ represent the connection weights between input and output nodes. *b* represents the excitation threshold of the neuron, that is, the bias. *y* is the output of the perceptron. The information transfer formula of the single-layer perceptron is expressed as follows:

$$y=\omega_{1}x_{1}+\omega_{2}x_{2}+b$$ (8)

**FIGURE 1:**
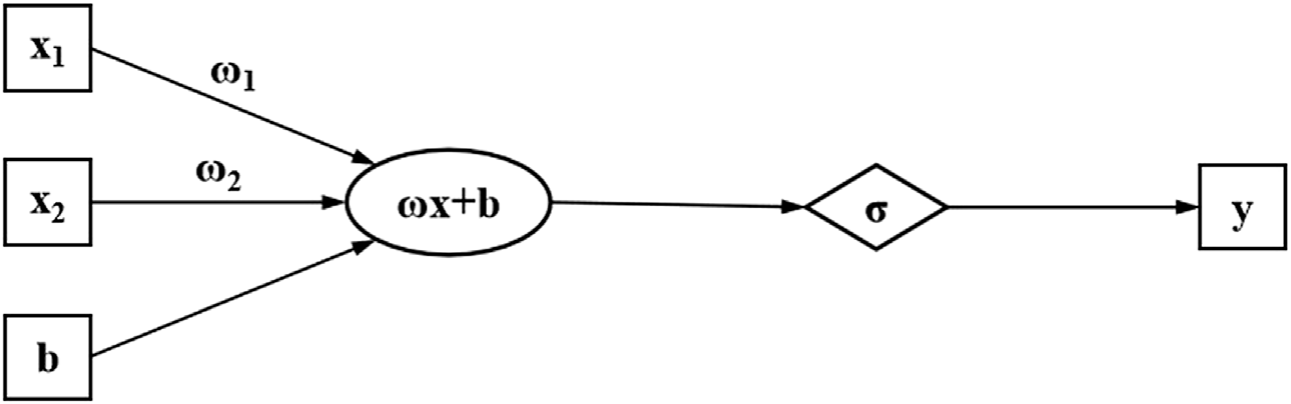
Schematic diagram of activation function.

The perceptron works by finding a linearly separable hyperplane separating the loaded data. What’s more, stronger data plane classification capabilities can be obtained by integrating multiple perceptron models. In the original perceptron model, the essence of the inter-layer transfer of the neural network is to perform a linear transformation of the data. This determines that a simple perceptron model can only solve linearly separable problems, but cannot deal with non-linear problems. Based on this, applying a non-linear activation function to the neuron node endows the neural network with the ability to solve linear inseparable problems. The non-linear activation function completes the linear transformation to fulfill output characteristics. As shown in Figure 1, the figure represents the non-linear modification of the linear transformation. The application of non-linear activation functions introduces non-linear factors into the neural network, allowing it to classify data planes using smooth surfaces. This enables the neural network to have superior data classification and regression capabilities.

### BP Neural Network

**FIGURE 2:**
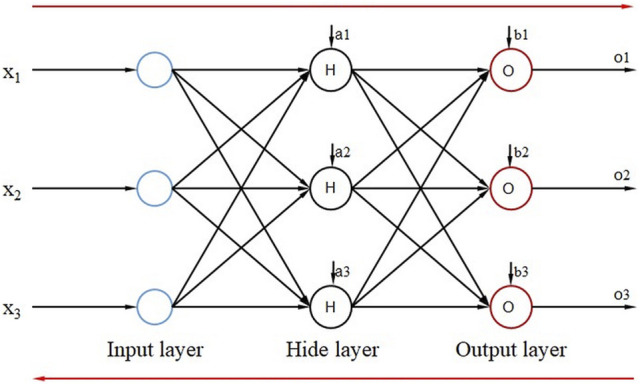
Schematic diagram of the topology structure of BPNN.

Assume that the number of nodes in the input, hidden, and output layers are *l*, *m* and *n*. The weights and biases from the input layer to the hidden layer and from the hidden layer to the output layer are in order ω_ij_, *ω*_*jk*_ and *a*_*j*_, *b*_*k*_, learning rate *η*. The excitation function is *g(x)*. The detailed steps of BPNN modeling are as follows:

BPNN is a multi-layer feedforward neural network based on an error back propagation algorithm. The principle is to use the common gradient descent algorithm and gradient search technology to minimize the error mean square error between the actual output value and the expected output value of the network in successive update iterations. The model topology of BPNN consists of an input layer, a hidden layer, and an output layer. Figure 2 shows a simple three-layer neural network. The input signal of the neural network acts on the output node through the hidden layer node and generates the output signal. By adjusting the connection weights of input nodes and hidden layer nodes, the connection weights and thresholds between the hidden layer and output nodes during the training, the deviation between the actual output and the expected output value decreases along the gradient direction. After repeated training and parameter adjustment, the network parameters (connection weights and thresholds) that match the minimum error are determined. The network parameters determined by the trained neural network process the input information of the input samples with similar characteristics and output the prediction information. Then the prediction result with the smallest error can be obtained.

Assume that the number of nodes in the input, hidden, and output layers are *l*, *m* and *n* respectively. The weights and biases from the input layer to the hidden layer and from the hidden layer to the output layer are *w*_*ij*_, *w*_*jk*_ and *a*_*i*_, *b*_*k*_. The learning rate is *η* and the excitation function is *g(x)*. The detailed steps of BPNN modeling are as follows:

(1) Network initialization. First, the weights and thresholds between the input and the hidden layers are initialized. Among them, the common activation function is the sigmoid function. Its function expression is,

$$f\left(x\right)=\frac{1}{1+e^{-x}}$$ (9)

(2) Data forward calculation. For the three-layer BPNN shown in Figure 2, the output sum of the hidden layer and the output layer *H*_*j*_ and *O*_*k*_ are respectively

$$H_{j}=g\left(\sum_{i=1}^{l}\omega_{ij}x_{i}+a_{j}\right)$$ (10)

$$O_{k}=g\left(\sum_{j=1}^{m}\omega_{jk}H_{j}+b_{k}\right)$$ (11)

(3) Error calculation

Assuming that *Y*_*k*_ is the expected output of the network, the error calculation formula is

$$E=\frac{1}{2}\sum_{k=1}^{n}e_{k}^{2}$$ (12)

$$e_{k}=Y_{k}-O_{k}$$ (13)

(4) Update of weights and biases. The weights and biases are updated through the back-propagation of the error to make the error function reach the minimum value, where the update formulas of the weights and biases are respectively

$$\{\begin{array}{c} \omega_{ij}=\omega_{ij}+\eta H_{j}\left(1-H_{j}\right)x_{j}\sum_{k=1}^{n}\omega_{jk}e_{k}\\ \omega_{jk}=\omega_{jk}+\eta H_{j}e_{k} \end{array}$$ (14)

$$\{\begin{array}{c} a_{j}=a_{j}+\eta H_{j}\left(1-H_{j}\right)x_{j}\sum_{k=1}^{n}\omega_{jk}e_{k}\\ b_{k}=b_{k}+\eta e_{k} \end{array}$$ (15)

Furthermore, we utilize mean squared error (MSE) to measure the accuracy of BPNN predictions. The formula for calculating the MSE is,

$$MSE=\frac{SSE}{l}=\frac{1}{l}\sum_{i=1}^{n}\omega_{i}\left(y_{i}-\overset{\wedge }{y_{i}}\right)$$ (16)

### The Training Process of BP Neural Network

The network training of BPNN includes the following processes: (1) Integrate the training data set. The training of BPNN requires large-scale data support, and the training samples need to contain both input data and label data. The accuracy of the data directly affects the prediction accuracy of the neural network. Data normalization is often necessary for the specific training to obtain similar weighting tendencies for different features. Normalization can reduce the computational complexity and speed up the convergence, and also facilitate the subsequent training optimization because the output of the activation function is 0-centered.

(2) Network structure design. The network structure aims to clarify the number of network layers, hidden layer nodes, the activation function between layers, the training and the loss function. As the network structure is determined, all data dimensions are established.
(3) Initialize the weights. The parameter update in the training of BPNN depends on the gradient descent algorithm (mainly including batch gradient descent (BGD) method, stochastic gradient descent (SGD) method, mini-batch gradient descent (MBGD) method, etc.). Before starting training with the gradient descent algorithm, the weights and biases in the network need to be initialized. To eliminate the symmetrical weights, many initialization methods are currently used to randomly initialize the weights of each layer. Too small initialization weight made the non-linear activation function lose its non-linear modification ability. On the contrary, too large an initialization weight causes the problem of gradient disappearance.
(4) Network training. BPNN is based on the back-propagation of the error between the network output and the label to update the weights and repeat the iterative calculation to make the loss function reach the expected desired value.

## Results

### CFD Data

We used the CFD data of immune cell transmigration as the training data of the BPNN model. Considering the limited effect of immune cell stiffness on cell motion and deformation and flow field in the transmigration simulation, it is not used as an input parameter of the neural network. This paper aims to construct a time-dependent prediction model for leukocyte transmigration to establish a model of immune cell movement and flow field. The data we use are all from the data obtained by our CFD simulation, including displacement parameters, cell deformation parameters, flow velocity, fluid lift, and drag parameters. We have fully investigated the hydrodynamic parameters of the environment in which immune cells migrate to ensure the accuracy of the data obtained by CFD. We performed blood flow *Re* of 0.1, 0.11, 0.12, 0.13, 0.14, 0.15, 0.16, 0.17, 0.18, 0.19, and 0.2 and *AR* of 0.5, 0.55, 0.6, 0.65, 0.7, 0.75, 0.8, 0.85, 0.9, 0.95, a total of 121 CFD simulations. Among them, the combination of *Re* and *AR* is (0.1, 0.5), (0.1, 0.55), (0.11, 0.5), (0.12, 0.5), (0.13, 0.5) and (0.14, 0.5). The impaction phenomenon is not included in the training. We will exclude 115 sets of CFD simulation data from these six sets of examples as the basic data set for training.

**FIGURE 3:**
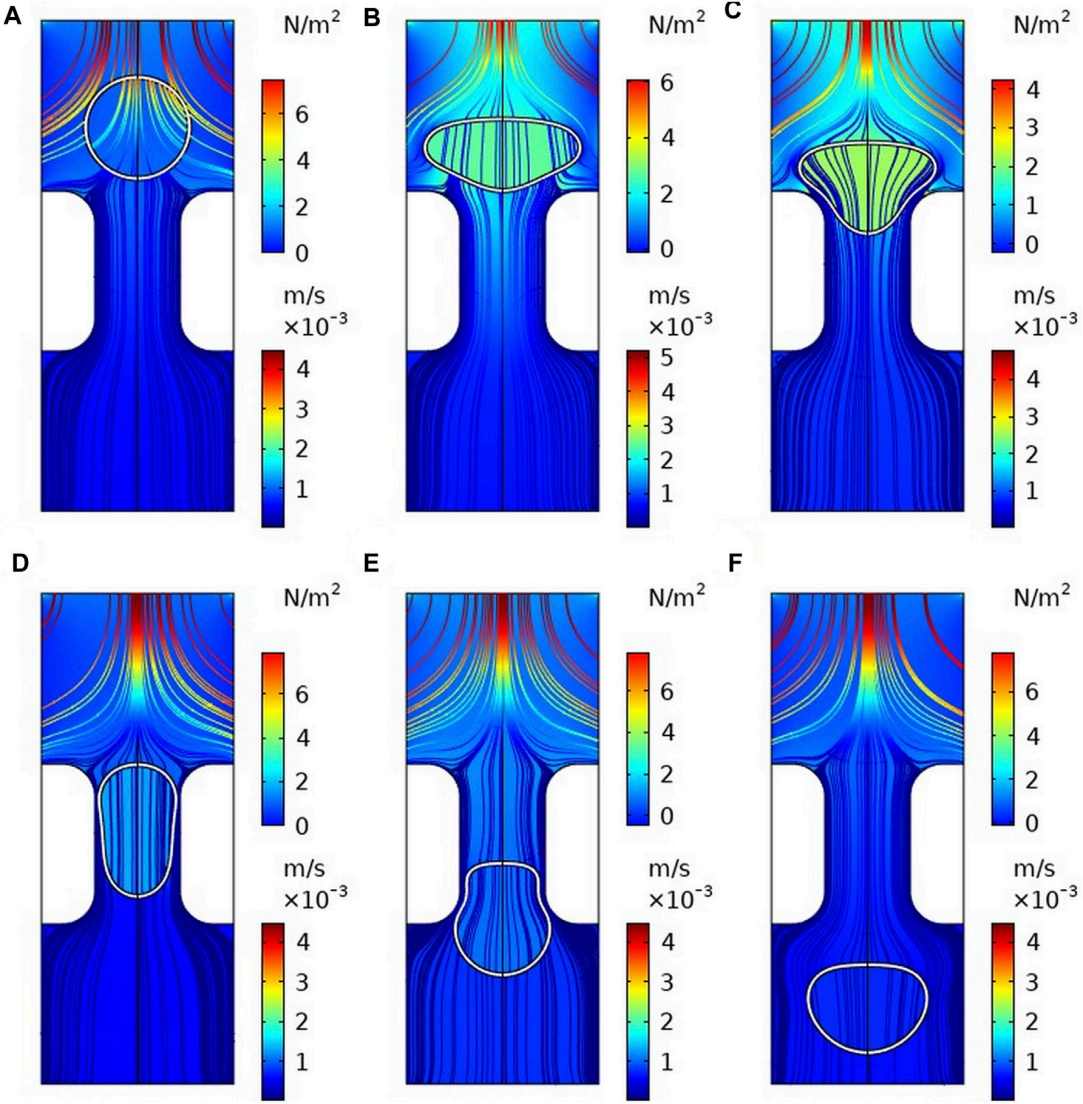
Flow field and pressure field distribution during cell transmigration **(A)** t = 0 s; **(B)** t = 0.002 s; **(C)** t = 0.01 s; **(D)** t = 0.05 s; **(E)** t = 0.1 s; **(F)** t = 0.15 s.

The 115 groups of *Re* and *AR* parameters in the basic data set are used as input data. The transmigration is the target vector to train the time-dependent leukocyte transmigration prediction model based on the BPNN model. In addition, we used 14,221 samples consisting of *Re*, *AR*, and cell downflow displacement parameters as a training dataset to train cell motion and flow field prediction models during transmigration. Among them, the target parameters are the cell deformation, the downstream velocity, the fluid lift, and drag forces. In order to test the accuracy of the model, 10 groups of *Re* and *AR* were used as (0.2, 0.53), (0.19, 0.57), (0.18, 0.63), (0.17, 0.67), (0.16, 0.73), (0.15, 0.77). New examples of (0.14, 0.83), (0.13, 0.87), (0.12, 0.93) and (0.11, 0.97) are used as test datasets to validate the time-dependent transmigration prediction model. The above parameter combination simulates the migration environment in which most immune cells are located and guarantees the generality of this study. At the same time, four groups of leukocyte transmigration CFD examples with *Re* and *AR* of (0.16, 0.73), (0.15, 0.77), (0.14, 0.83) and (0.13, 0.87) were used to test the accuracy of the prediction model of the transmigration. The hydrodynamic simulation still uses the leukocyte transmigration CFD model shown in Figure 3.

### Time-Dependent Prediction of Leukocyte Transmigration Based on BP Neural Network Model

Many studies have shown that a 4-layer neural network with only two hidden layers can predict most regular data matrices accurately. The input parameters of the time-dependent leukocyte transmigration prediction model are two vectors, *Re* and *AR*, so the number of neurons in the input layer is 2. To avoid the long training time caused by many nodes, we draw on the empirical formula for node selection in the hidden layer,

$$\sum_{n=0}^{M}C_{I}^{n}>h$$ (17)

**FIGURE 4:**
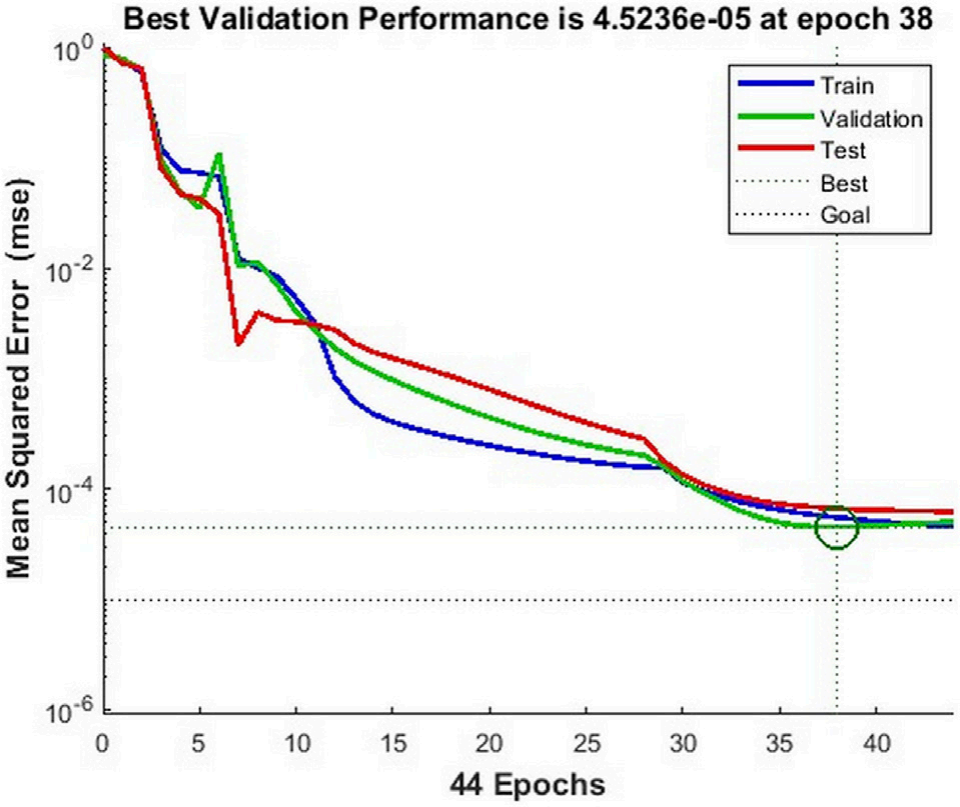
Training performance of the time-dependent model for leukocyte transmigration.

Where *M* is the number of nodes in the input layer, namely 2. *I* is the number of hidden layer nodes to be used. *h* is the number of training samples of the BP model. After calculation, the number of nodes in the hidden layer should not be less than 15. Therefore, we use two-layer hidden layers with 5 and 10 nodes, respectively, to construct the time-dependent cell transmigration prediction model. The activation function from the input layer to the hidden layer and the hidden layer adopts the tansig function, and the activation function from the hidden layer to the output layer adopts the purelin function. In addition, the trainlm function is used as the training function of the BP neural network. Meanwhile, the maximum training times, training target and learning rate are 1,000, 1 × 10^–5^ and 0.03, respectively. Figure 4 shows the training performance of the time-dependent leukocyte transmigration prediction model. The model achieves the best verification performance when the number of training times is 38, and its value is 4.524 × 10^–5^.

**FIGURE 5:**
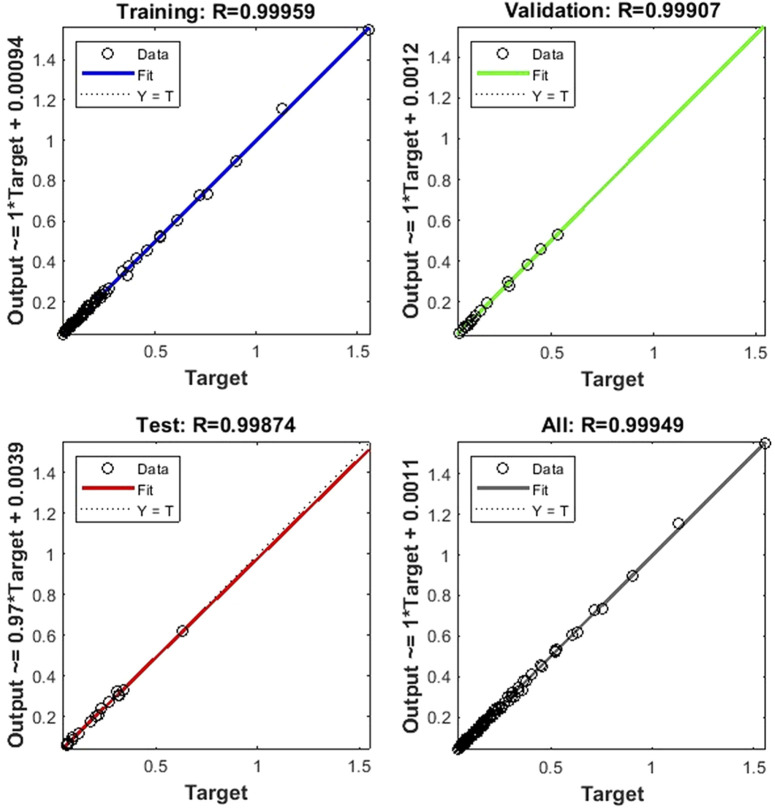
Fitting curve of time-dependent leukocyte transmigration model.

Based on the default partitioning method, the BPNN model randomly assigns 115 samples to the training set, validation set and test set according to the ratio of 60%, 20%, and 20%. Figure 5 shows that the regression accuracies on the training set, validation set, test set, and total dataset reached 0.99959, 0.99907, 0.99874, and 0.99949, respectively. This further proves the prediction robustness of the BPNN model.

To test the prediction accuracy of the leukocyte transmigration time prediction model, we predicted the transmigration time of 10 groups of cases in the test data set different from the training samples and compared them with the CFD results. We use the coefficient of determination as a criterion to analyze the deviation between the BPNN and CFD simulation and further measure the robustness of the mechanical prediction system. The formula for calculating the coefficient of determination is:

$$R^{2}=\frac{\sum {\left(\hat{y_{j}}-\overset{-}{y}\right)}^{2}}{\sum {\left(y_{j}-\overset{-}{y}\right)}^{2}}$$ (18)

Where $\hat{y_{j}}$ is the predicted value of BPNN and $\overset{-}{y}$ is the average value of the real value of the CFD simulation, *y*_*j*_ is the true value of the CFD simulation. The interval range of the coefficient of determination is [0, 1], and the closer to 1, the better the prediction effect.

**FIGURE 6:**
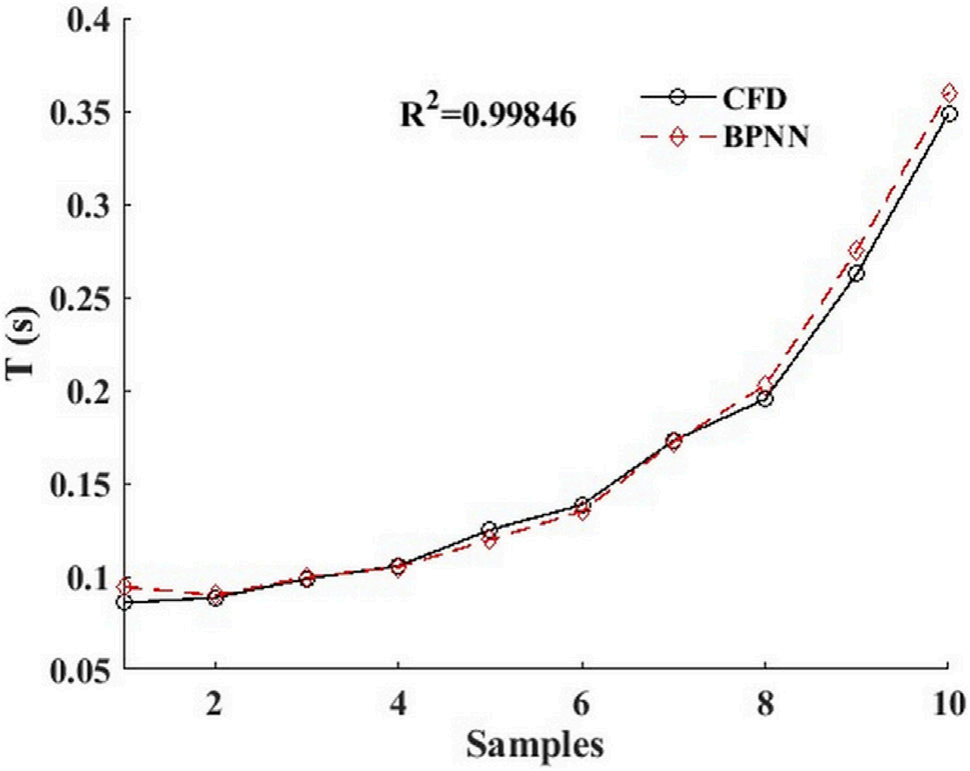
BPNN prediction value and CFD real value of 10 groups of calculation examples based on time-dependent leukocyte transmigration prediction mode.

**FIGURE 7:**
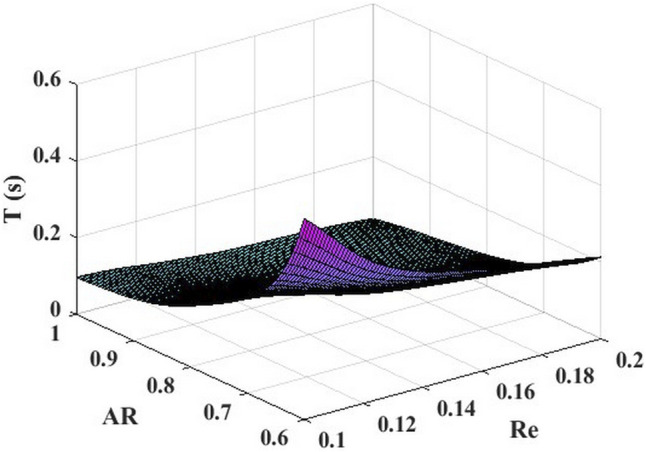
Time-dependent leukocyte transmigration prediction model based on BP neural network.

Figure 6 shows the comparison between the predicted time-dependent of BPNN and the time-dependent CFD for 10 groups of leukocyte transmigration examples from the *Re* interval of [0.1, 0.2] and the *AR* interval of [0.5, 1]. The results show that the predicted value in the middle region of the *AR* interval is in good agreement with the true value and slightly worse at the two ends. The *R* ^*2*^ was 0.99846, indicating that the leukocyte transmigration prediction model had excellent prediction accuracy. Based on this, we constructed a BPNN-based time-dependent leukocyte transmigration prediction model a three-dimensional map of the predicted leukocyte transmigration time in the *Re* interval [0.1, 0.2] and *AR* interval [0.6, 1] (Figure 7). As shown, the leukocyte transmigration time increased significantly with the decrease of *Re*. Compared with *Re*, the reduction of *AR* has a more prominent effect on prolonging the time-dependent leukocyte transmigration. Under the dual influence of *Re* and *AR*, the increase in leukocyte transmigration time was more pronounced and manifested as a sharp upward curl on the map.

### Prediction of Leukocyte Transmigration Based on BP Neural Network Model

The prediction model can predict the time-dependent leukocyte transmigration in specific *Re* and *AR* intervals. However, the morphological changes of cells during transmigration and the changes in the surrounding flow field remain unclear. The robustness of the BPNN model aided to construct a leukocyte transmigration prediction model. As mentioned above, 14,221 samples including *Re*, *AR*, cell down-flow displacement, cell deformation, down-flow velocity, received fluid lift and drag parameters were used as the training dataset to train the BPNN model. The data matrix containing *Re*, *AR* and cell forward displacement is used as input data to establish the corresponding relationship with the target data. The target data vectors are *R*_*max*_, *V*_*y*_, *F*_*l*_ and *F*_*d,*_ respectively. Using Eq. 14 to calculate the number of neurons in the hidden layer, we use 50 nodes to fill the hidden layer. We construct a two-layer hidden layer with 20 and 30 nodes, respectively. The selection of activation and training functions is the same as the leukocyte transmigration time-dependent prediction model. At the same time, the determination coefficient of Eq. 11 is still used to measure the prediction accuracy of the BPNN prediction model.

### Cell Motion Prediction

**FIGURE 8:**
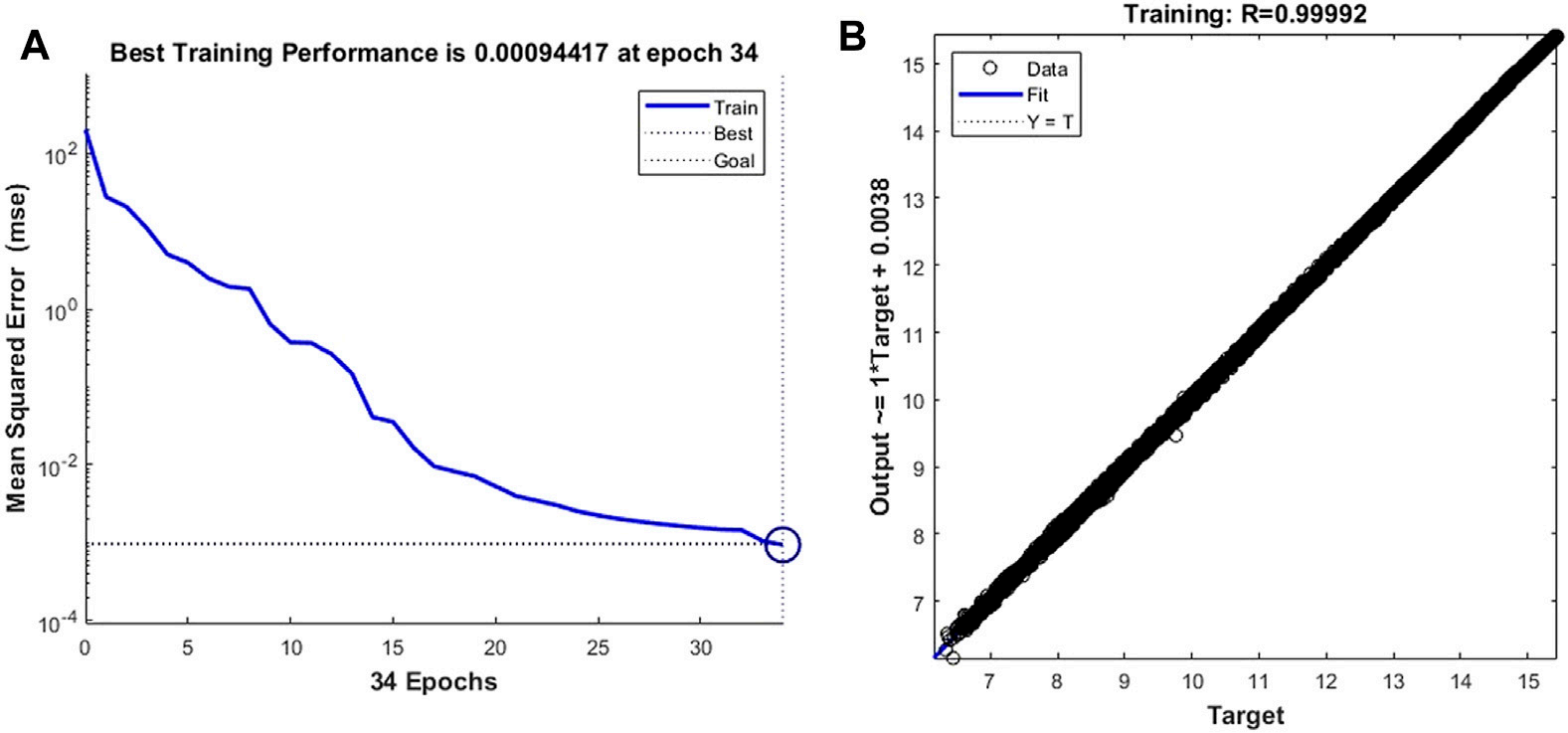
Training performance and fitting curve of the leukocyte transmigration prediction model for cell deformation prediction **(A)** Training performance; **(B)** Fitting curve.

First, we train the neural network with cell deformation parameters as target vectors. Unlike the setting threshold of the time-dependent prediction model of leukocyte transmigration, the better prediction accuracy can be obtained by setting the training target as 10^–3^. As shown in Figure 8A, the prediction model achieves the best training performance when the number of training times is 34, and its value is 0.00094417. At the same time, a fitting accuracy of 0.99992 was obtained in training on the overall training set with a sample size of 14,221 (Figure 8B).

**FIGURE 9:**
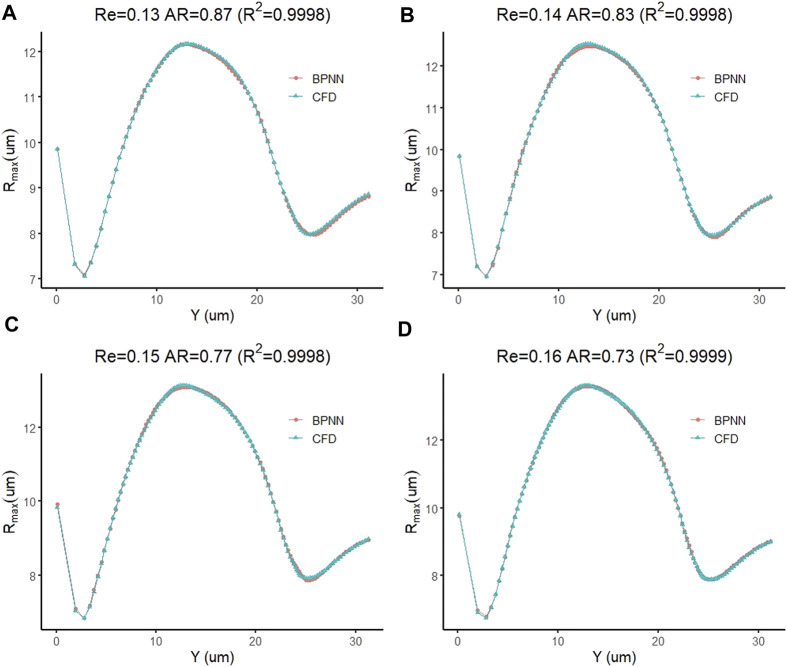
Prediction accuracy test of a leukocyte transmigration prediction model for cell deformation prediction.

Next, we use the cell deformation prediction model to predict the cell deformation during the transmigration for the four new examples mentioned above. Their Re and *AR* are set to (0.16, 0.73), (0.15, 0.77), (0.14, 0.83), and (0.13, 0.87), respectively. Figure 9A–D compares the predicted cell deformation values and the CFD values of the four groups of calculations, respectively. The figure shows the deformation prediction of the cell forward displacement by the prediction model is consistent with the CFD results. The coefficients of determination, which measure the degree of agreement between the BPNN predictions of the four groups of examples and the real solutions of CFD, are 0.9998, 0.9998, 0.9998, and 0.9999, respectively, showing an excellent prediction effect.

**FIGURE 10:**
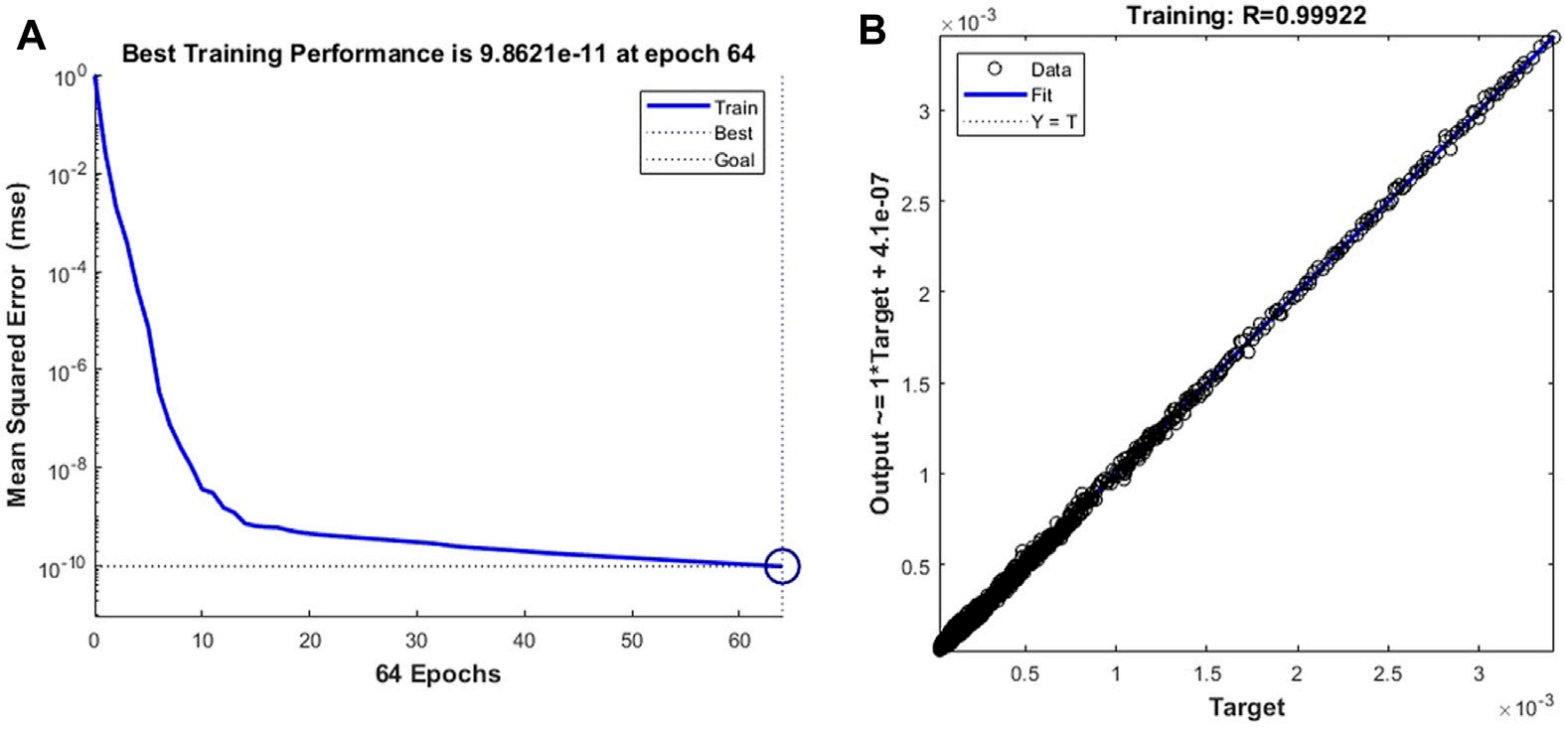
The training performance and fitting curve of the leukocyte transmigration prediction model used for cell downflow velocity prediction **(A)** Training performance; **(B)** Fitting curve.

**FIGURE 11:**
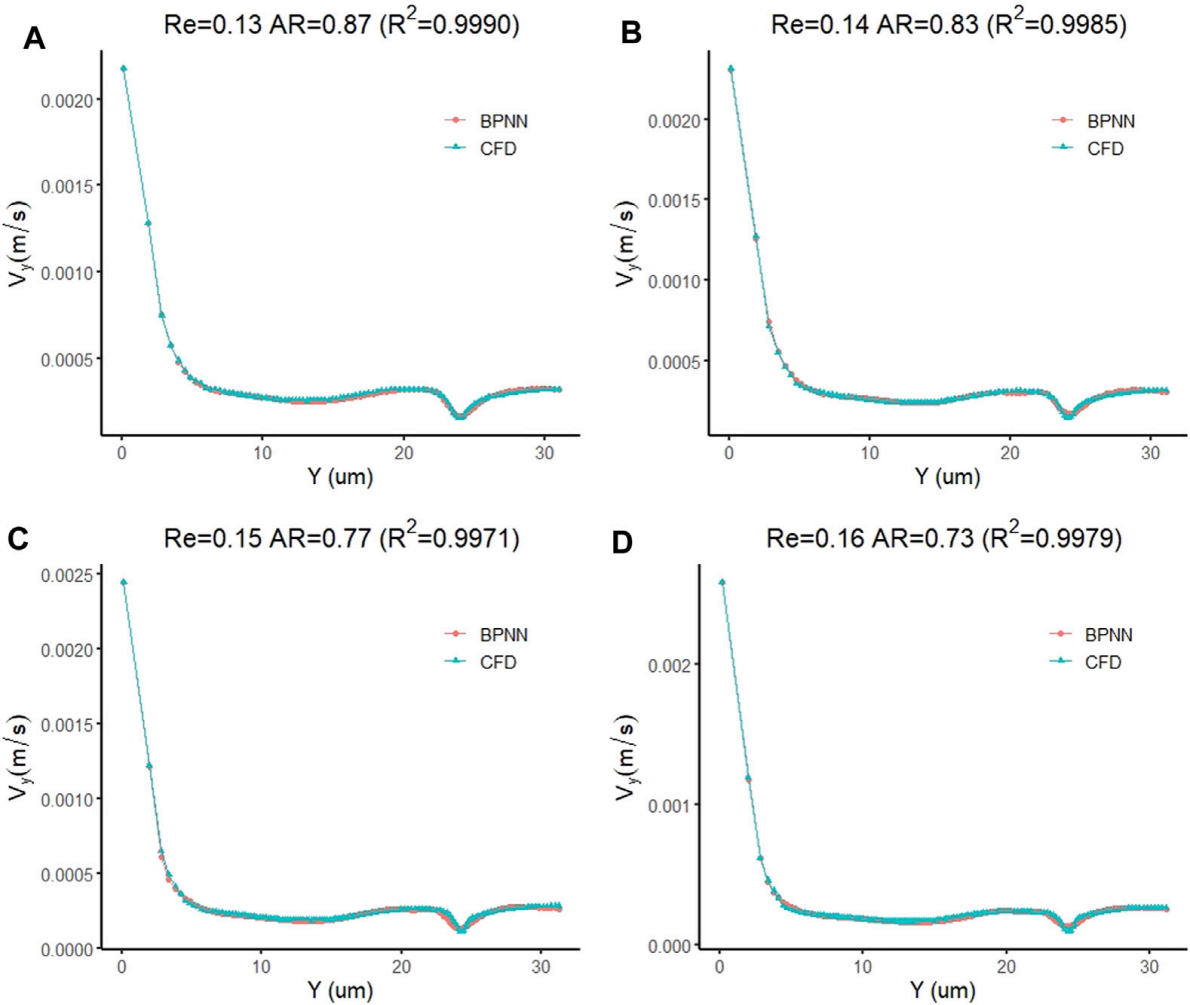
Prediction accuracy test of the leukocyte transmigration prediction model used for cell forward velocity prediction **(A)** Re = 0.13, AR = 0.87; **(B)** Re = 0.14, AR = 0.83; **(C)** Re = 0.15, AR = 0.77; **(D)** Re = 0.16, AR = 0.73.

By changing the target vector of the prediction model to the velocity vector of the cell forward movement, we constructed a model for predicting the leukocyte transmigration speed. Due to the increased degree of non-linearity between the input data and the target vector, the threshold for the training target was set to 10^–10^. The training performance of the model and the data fitting curve are shown in Figure 10A,B. When the number of training times reaches 64, the BPNN model obtains the best training performance with a value of 9.862 × 10^–11^. At the same time, the *R* value of the training data fitting reached 0.99922, indicating a good mapping relationship between the input data and the output data in the forecast model. Subsequently, we tested the established cell forward velocity prediction model. Using the same 4 datasets, the good predictive ability of the forecasting model is revealed. Figure 11A–D all show that the predicted value of BPNN can well fit the CFD solution, and the coefficients of determination of the closeness between the predicted curve and the real curve are 0.9990, 0.9985, 0.9971 and 0.9979.

### Prediction of Cell Lift and Drag Force

**FIGURE 12:**
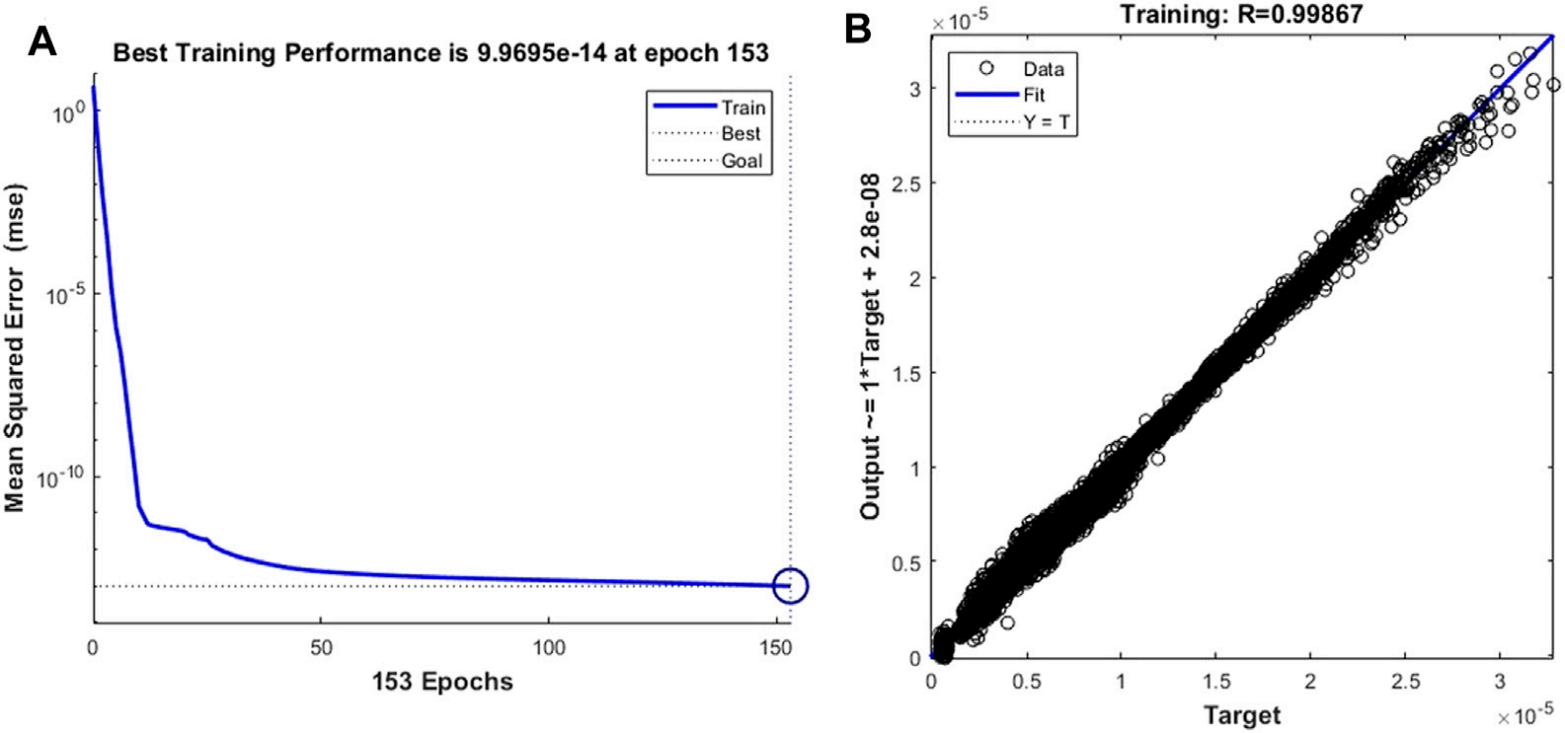
Training performance and fitting curve of the leukocyte transmigration prediction model used for the prediction of fluid lift forces on cells **(A)** Training performance; **(B)** Fitting curve.

Based on the same network skeleton of the leukocyte transmigration prediction model, we replace the trained target vector with the cell fluid lift forces to calculate the accuracy of the prediction model in predicting the cell fluid lift forces. Since the non-linearity of the data further increases, the training objective and minimum gradient threshold are set to 1 × 10^–13^ and 1 × 10^–10^, respectively, to obtain better prediction accuracy. Figure 12A shows that the prediction model achieved the best training performance when the training order reached 153, and its value was 9.970 × 10^–14^. At the same time, the fitting curve of the model achieved an accuracy of 0.99867 (Figure 12B).

**FIGURE 13:**
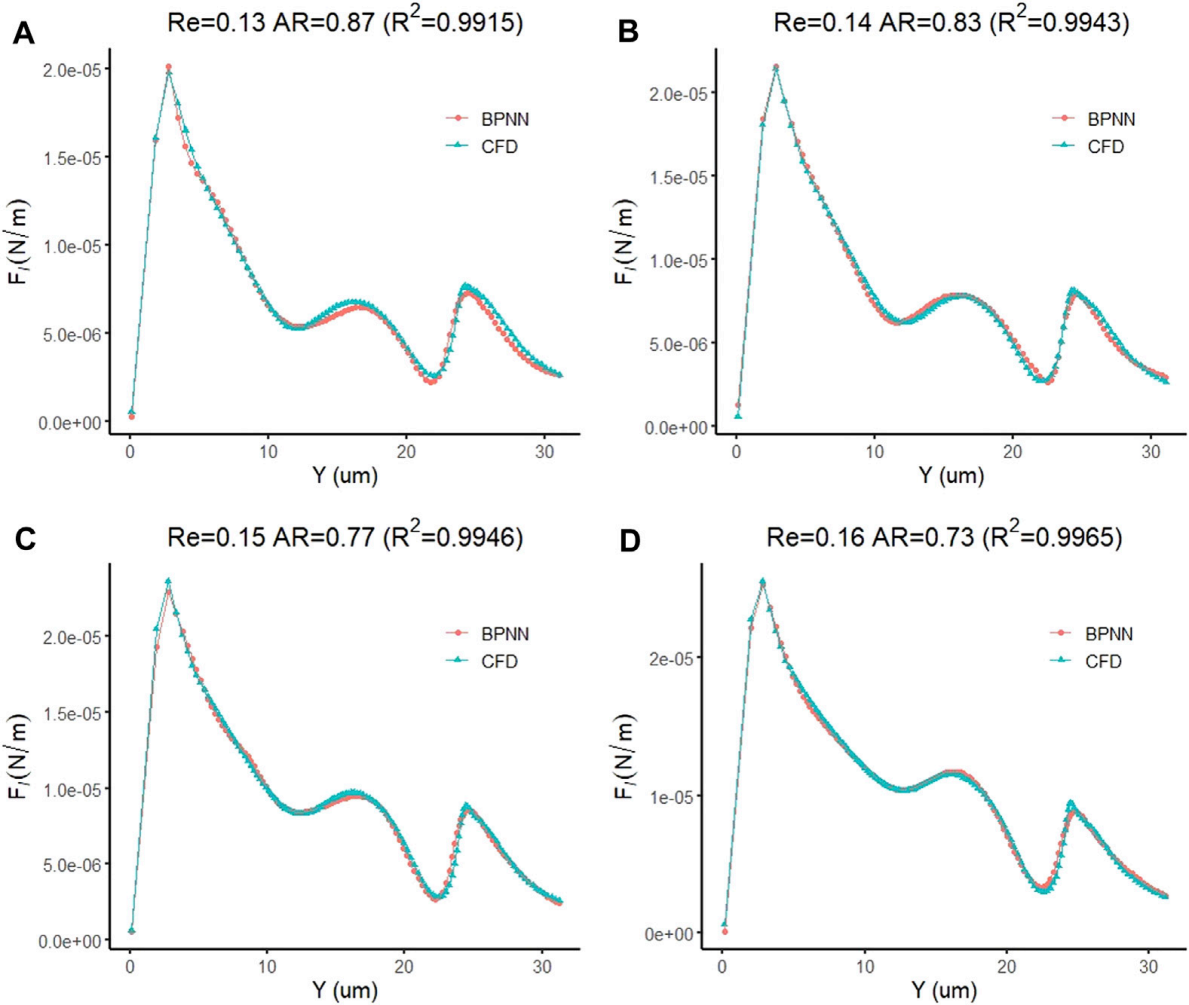
Prediction accuracy test of the leukocyte transmigration prediction model for the prediction of fluid lift forces on cells **(A)** Re = 0.13, AR = 0.87; **(B)** Re = 0.14, AR = 0.83; **(C)** Re = 0.15, AR = 0.77; **(D)** Re = 0.16, AR = 0.73.

Similar to the prediction of cell deformation and downstream motion velocity, we also found that the prediction model of the transmigration is highly accurate in predicting the fluid lift force on the cell. Figure 13A–D of show the prediction curves of the prediction model for the fluid lift forces on cells in the four new cases, respectively. The results show that the predicted curve is consistent with the CFD curve, and the difference between the predicted and actual values at each data point is very small. The coefficients of determination of the predicted curve and the real CFD curve in the four groups of examples are 0.9915, 0.9943, 0.9946 and 0.9965, respectively, showing excellent prediction performance.

**FIGURE 14:**
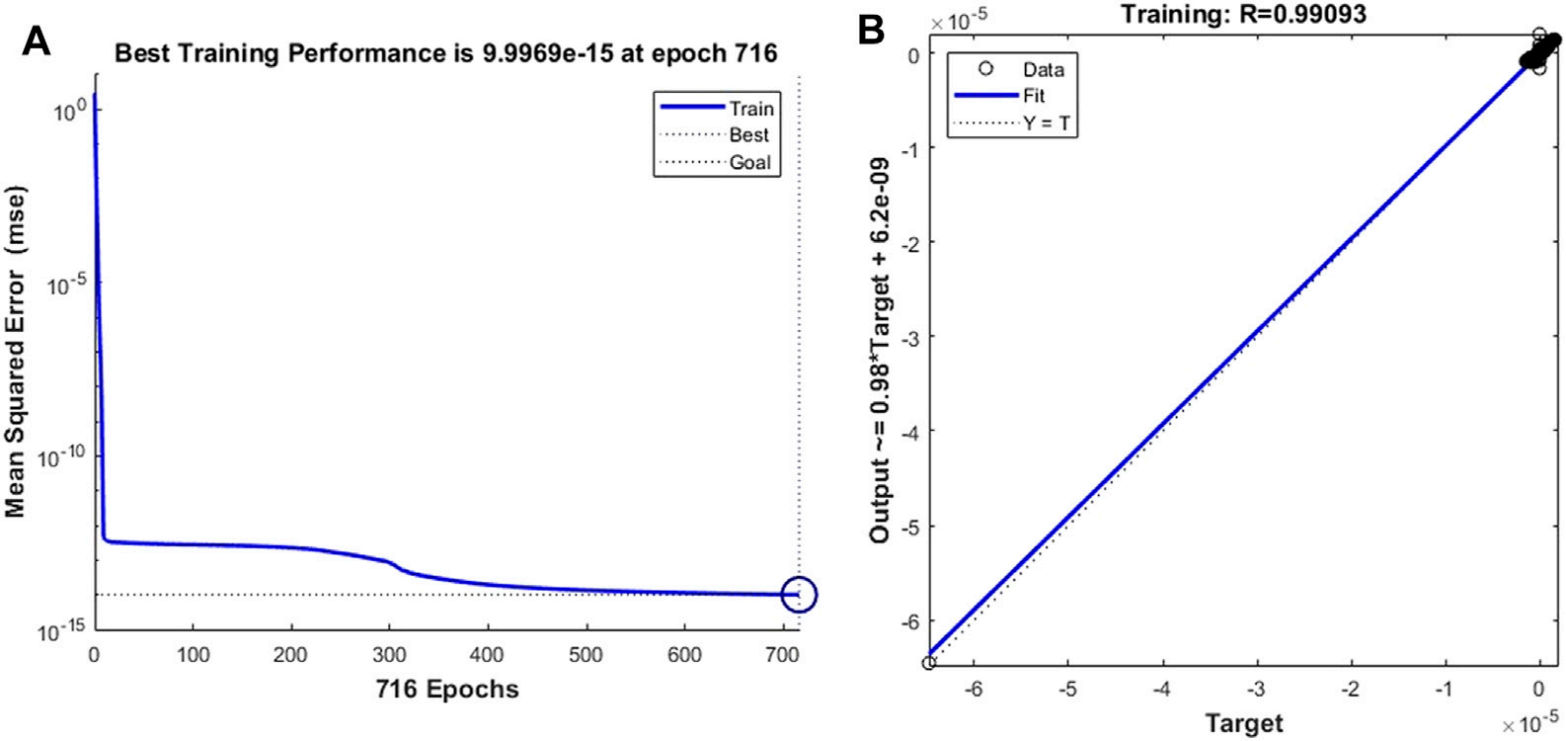
Training performance and fitting curve of the leukocyte transmigration prediction model used for the prediction of drag force to cells **(A)** Training performance; **(B)** Fitting curve.

**FIGURE 15:**
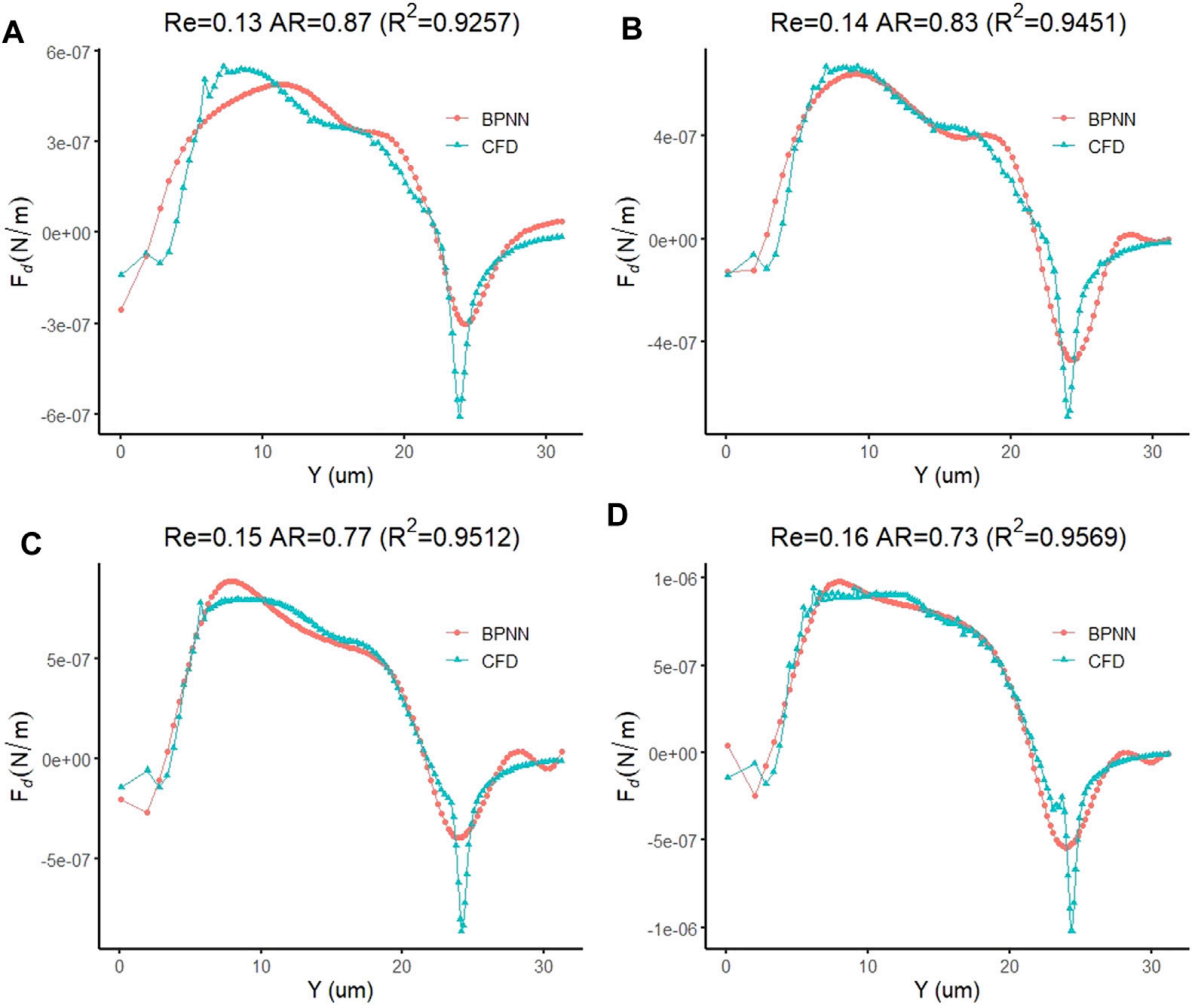
Prediction accuracy of the leukocyte transmigration prediction model for the prediction of fluid lift forces on cells **(A)** Re = 0.13, AR = 0.87; **(B)** Re = 0.14, AR = 0.83; **(C)** Re = 0.15, AR = 0.77; **(D)** Re = 0.16, AR = 0.73.

Finally, we replaced the target vector of the leukocyte transmigration prediction model with the drag force of the cell to observe the prediction ability of the model to the drag force. After adequate iterations were implemented, it was found that the training model with the training target and the minimum gradient threshold set to 1 × 10^–14^ and 1 × 10^–12^, respectively, could maintain a good prediction of the target. When the number of training times reaches 716, the model has the best training performance with a value of 9.997 × 10^–15^ (Figure 14A). At this point, the fitting parameter of the training data reached 0.99093 (Figure 14B). Based on the same test case, we analyzed the prediction accuracy of the prediction model for the drag force of cells during the transmigration. Figure 15A–D show the drag force prediction curves and CFD curves of different examples, respectively. The prediction accuracies of these examples are 0.9257, 0.9451, 0.9512 and 0.9569, respectively. The entire computation process, including CFD and BPNN, is shown in Figure 16. The prediction models have significant discrepancies in the prediction of the drag force in the initial cell movement stage, the pore entry stage and the pore exit stage. The model maintained a high prediction accuracy for drag force in other periods.

### Computational Efficiency Comparison

Compared with traditional CFD simulation methods, we found that BPNN greatly shortens the simulation time of the model and maintains high accuracy under the condition of sufficient sample size. Once the network model is trained, BPNN can instantaneously predict the time-dependent transmigration of cells. The computer configuration for this article is (Intel(R) Core(TM) i5-4570 3.2 GHz RAM 8G). Based on the same computing resources, the training time of the BP model is less than 10 min in total. In contrast, the total time required for establishing and training the BP model is comparable to that of a CFD example. This fully reflects the punctuality and efficiency of BPNN forecasting.

## Discussion

Immune cell infiltration and is of great concern in the biomedcine (Shen et al., 2020). Traditional CFD methods are often accompanied by complex and time-dependent modeling and calculations (Khaleghi et al., 2021). Based on this, many leukocyte transmigration CFD simulation data obtained in the previous paper, we tentatively established a time-dependent prediction model of the transmigration. The cellular transmigration time-dependent prediction model can predict the time it takes for cells to pass through the pores in the vascular environment in specific *Re* and *AR* intervals. On this basis, the leukocyte transmigration model can predict the cell movement and flow field changes during the transmigration with extremely high accuracy. Both prediction models have a double hidden layer structure, and the total number of hidden layer nodes is 15 and 50, respectively. The time-dependent leukocyte transmigration prediction model only has two input parameters, *Re* and *AR*. The input data of the leukocyte transmigration prediction model also includes the cell forward displacement parameter. Based on the constructed model, we selected 10 time-dependent samples of leukocyte transmigration and 4 examples of leukocyte transmigration in the training *Re* and *AR* intervals to test the prediction accuracy of the two prediction models and obtained superior predictions Effect. Compared to traditional CFD techniques, the predictive capability of the BPNN model significantly highlights its potential applications in fluid mechanics (Wong and Kim, 2018).

With the rise of the technology, many algorithms have emerged that incorporate physical models (Jiang et al., 2019; Liu et al., 2022; Liu et al., 2022). In this regard, the neural network family to which BP belongs plays an important role (Wong and Kim, 2018). Earlier studies have simply used neural network algorithms to improve the accuracy of data processing (Rediniotis and Vijayagopal, 1999). For example, Wang et al. applied the artificial neural network based on BP theory to the data processing of the five-hole probe in the fluid experiment, and put forward the conclusion that the accuracy and reliability of the prediction results are better than the linear three-dimensional interpolation method. Then, the predictive capabilities of neural networks, which can maintain high accuracy and rapidity, are gradually applied to fluid mechanics (Rediniotis and Vijayagopal, 1999; Fan et al., 2003; Cheng et al., 2020). For example, the BP model can effectively predict the displacement and dominant frequency of the vortex-induced vibration of flexible cylinders commonly found in engineering. But the neural network has a significant disadvantage, that is, the successful training of the network needs to rely on a considerable number of samples (Liu et al., 2015; Chen, 2017). In this regard, other algorithms can better address this problem (Jiang et al., 2019; Sun et al., 2022; Wang and Cao, 2029). The reason for abandoning the large-scale physical models and choosing the neural network model is primarily because of its efficient and fast simulation and prediction capabilities (Li and Peng, 2007; Kang and Cho, 2014). Like most forecasting models, the two forecasting models established in this paper based on the BPNN model show the instantaneousness and high accuracy of forecasting.

A sufficiently trained neural network model also can guide engineering design, such as the rational planning of the airfoil design process by predicting the leading edge pressure distribution of a hybrid airfoil (Yao and Sung et al., 2018; Sekar et al., 2019; 廖鹏与姚磊江等, 2019). What’s more, an active flow controller incorporating deep reinforcement learning neural networks significantly reduces lift and drag fluctuations in the flow around a cylinder (Haryanto et al., 2014; Peng et al., 2020; Tang et al., 2020). Because of the wide application of BPNN model in fluid mechanics, we integrated the leukocyte transmigration CFD dataset to establish a time-dependent leukocyte transmigration prediction model. We randomly predicted the CFD results of leukocyte transmigration models for several groups of different *Re* and *AR* using established two-class prediction models. Compared with the real CFD results, the prediction model showed robustness in both the time-dependent transmigration and the changes of cell motion and flow field during the transmigration.

This paper mainly focuses on the processing of leukocyte migration data obtained by CFD simulation using BPNN technology, and fully demonstrates the advantages of artificial neural networks for the prediction of immune cell perforation compared with traditional CFD simulation after having a certain amount of data in the early stage. This advantage is mainly reflected in forecasting speed and reliable accuracy. The effects of biochemical factors on leukocyte migration were significantly different from mechanical factors on leukocyte migration. The adhesion and migration of immune cells are crucial in the body immunity and host defense. Integrins on the surface of immune cells are the core molecules regulating immune cell adhesion and migration. The main scientific question to explore the influence of biochemical factors is how immune cells sense changes in the extracellular microenvironment, thereby controlling tissue-specific migration of immune cells by regulating integrin function. It is more of an on-off regulation. Migration often occurs because cells sense signals from the outside world, such as white blood cells sensing abnormal proteins released by bacteria. The cell then turns on a switch inside itself, initiating the migration process. The mechanical factor focuses on the fluid environment in which the immune cells in the blood vessels are located and the forces they experience during migration, such as the fluid environment characterized by the two parameters *Re* and *AR* in this paper.

We performed numerical simulations of the model based on the fine, finer, and ultrafine meshing methods, respectively, to verify the reliability of our CFD results. Relevant content has been added to the revised manuscript. The numbers of meshes included in the numerical model under the fine, finer and ultrafine meshing are 8522, 21,237 and 55,245 meshes, respectively. As shown in Supplementary Figure S1A, the time-Rmax curves of the three meshing methods have the same trend. However, the resulting finer distribution of data points differs slightly from the other two meshing methods. The finer and ultra-fine results can fit well. Considering the increasing demand for computing resources brought about by the increase in grids, we use a finer grid division method for subsequent simulations. As shown in Supplementary Figure S1B, the finer mesh division method locally refines the fluid-structure interaction boundary based on the physical model of cell perforation based on different physical field distributions. The mesh model based on this meshing method can better meet the calculation requirements of the physical model with large non-linear characteristics to ensure that the results are reliable enough.

Overall, our results demonstrate the feasibility and robustness of BPNN in prediction studies of leukocyte transmigration. The strong generalization ability of the neural network also provides a technical guarantee for a comprehensive and complete prediction model of cell infiltration in the future. Compared with the traditional CFD technology, the neural network model is easy to implement because it does not require an explicit fitting function. At the same time, the prediction time of this method also significantly shortens the simulation time based on traditional physical models.

## Data Availability

The raw data supporting the conclusion of this article will be made available by the authors, without undue reservation.

## Supplementary Material

Supplementary Figure S1 Mesh independence verification and ultra-fine meshing **(A)** Time-Rmax curve under relatively fine, finer, and ultrafine meshing; **(B)** Based on the ultra-fine meshing method global and local meshing of the cell perforation physical model.
