# Supplementary material for: A Transendothelial Leukocyte Transmigration Model Based on Computational Fluid Dynamics and BP Neural Network
Source: Front Bioeng Biotechnol. 2022 Jun 21;10:881797. doi: 10.3389/fbioe.2022.881797 (PMC9253467; doi:10.3389/fbioe.2022.881797)
Supplement: Supplementary file 1 [file Table1.DOCX]

| **Parameters** | **Description** | **Value(units)** |
| --- | --- | --- |
| **r_c_** | Cell radius | 5 μm |
| **t_cm_** | Cell membrane thickness | 0.4 μm |
| **r_cp_** | Cytoplasmic radius | 4.6 μm |
| **D** | Computational domain width | 18 μm |
| **L** | Computational domain length | 46 μm |
| **d** | Pore width | 5 ~ 10 μm |
| **E** | Cell membrane Young's modulus | 50 ~ 500 Pa |
| **ν** | Poisson's ratio | 0.25 |
| **ρ_cm_** | Cell membrane density | 1000 kg/m^3^ |
| **ρ_cp_** | Cytoplasm density | 1000 kg/m^3^ |
| **ρ_p_** | Plasma density | 1000 kg/m^3^ |
| **μ_cp_** | Cytoplasm kinematic viscosity | 0.0008 Pa·s |
| **μ_p_** | Plasma kinematic viscosity | 0.0008 Pa·s |
| **Re** | Reynolds number | - 1. ~ 0.2 |
| **AR** | Aspect ratio | 0.5 ~ 1 |
