# Supplementary figures and images for: A Transendothelial Leukocyte Transmigration Model Based on Computational Fluid Dynamics and BP Neural Network
Source: Front Bioeng Biotechnol. 2022 Jun 21;10:881797. doi: 10.3389/fbioe.2022.881797 (PMC9253467; doi:10.3389/fbioe.2022.881797)

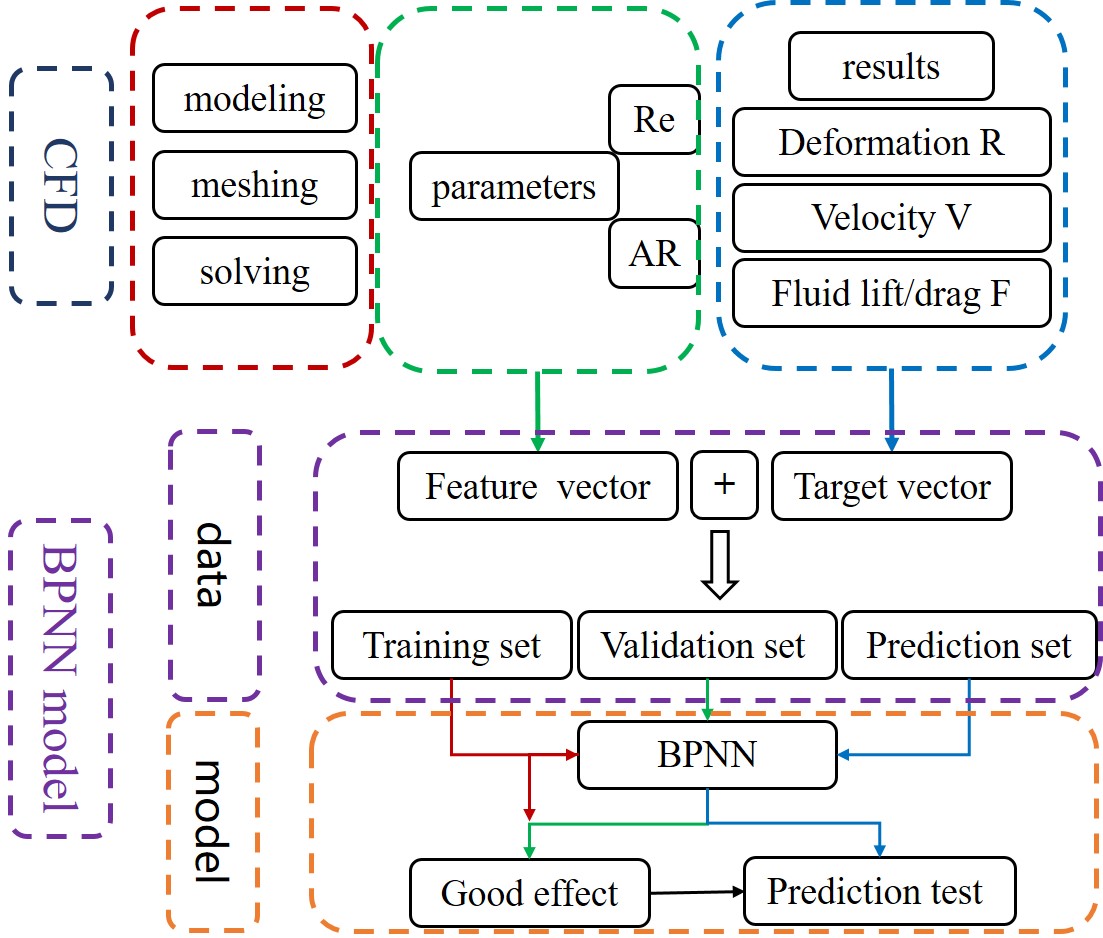

Supplement: Supplementary file 2 [file Image1.JPEG]

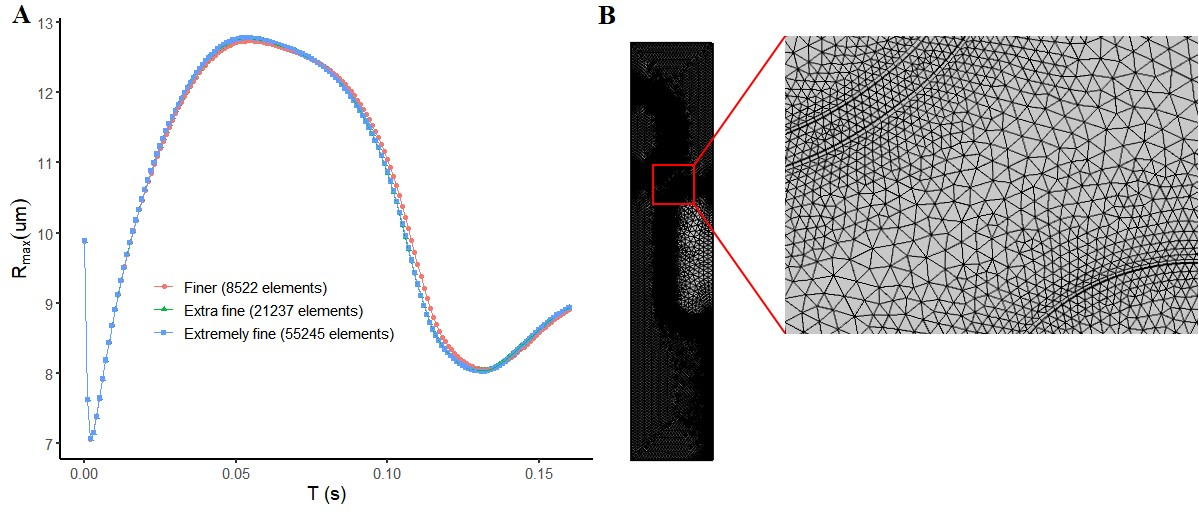

Supplement: Supplementary file 3 [file Image2.TIF]
